# Prehatching temperatures drive inter-annual cohort differences in great tit metabolism

**DOI:** 10.1007/s00442-022-05126-7

**Published:** 2022-02-17

**Authors:** Juli Broggi, Esa Hohtola, Kari Koivula, Seppo Rytkönen, Jan-Åke Nilsson

**Affiliations:** 1 Department of Biology, Section of Evolutionary Ecology, University of Lund, 223 62 Lund, Sweden; 2 Estación Biológica de Doñana (CSIC), Av. Américo Vespucio 26, 41092 Sevilla, Spain; 3 Departamento de Ecología Evolutiva, Museo Nacional de Ciencias Naturales - CSIC, C/José Gutiérrez Abascal 2, 28006 Madrid, Spain; 4 Ecology and Genetics Research Unit, Faculty of Science, University of Oulu, P.O. Box 8000, FI-90014 Oulu, Finland

**Keywords:** Basal metabolic rate, Breeding vs wintering, Carry-over, Early developmental conditions, Transgenerational plasticity

## Abstract

Basal metabolic rate (BMR) constitutes the lowest metabolic rate in a resting animal and is, therefore, considered to reflect the energetic cost of maintenance in endotherms. BMR is a reversible plastic trait that changes with environmental and ecological circumstances, albeit being heritable and susceptible to selection. Inter-individual variation within populations of small birds is substantial, and while many of the drivers of such variation have been identified, many remain unexplained. We studied winter BMR variation of juveniles over a 15-year period in a wild population of great tits Parus major at the northern border of their distribution. BMR during winter consistently changed between years, even after controlling for environmental factors, suggestive of a non-reversible developmental plasticity shaping the adult metabolic phenotype. BMR in cohorts of wintering great tits varied among winters as a response to minimum ambient temperatures experienced early in life, during the prehatching period. This developmental plasticity might be adaptive if temperatures experienced by growing embryos would metabolically prime them to an environment that they will likely encounter in future life. However, in line with a more unpredictable future climate, the risk of phenotype-environment mismatch is likely to lead to certain cohorts being poorly adapted to prevailing winter conditions, resulting in wider annual fluctuations in population size.

**Supplementary Information:**

The online version contains supplementary material available at 10.1007/s00442-022-05126-7.

## Introduction

Small birds acclimatize to winter conditions by improving their cold-endurance and thermogenic capacity, which correlates with a seasonal increase of internal energy reserves and basal metabolic rate (BMR) (Swanson 2010). BMR constitutes the lowest metabolic rate in a resting animal and is thereby considered to reflect physiological maintenance costs. In general, selection should keep the maintenance costs as low as possible (Swanson et al. 2017), although there might be individual variation in the optimal level of investment in maintenance (Petit and Vézina 2014a). Maintenance costs constitute a major part of energy expenditure during winter nights in resident birds particularly at high latitudes (Broggi et al. 2004), which often correlates with the individual thermogenic capacity (Broggi et al. 2004; Swanson 2010; McKechnie and Swanson 2010). However, this relation is not straight forward as BMR and maximal energy expenditure for thermogenesis, representing cold endurance and thermogenic capacity, are not functionally linked (Petit et al. 2013; Petit and Vézina 2014a). Variation in BMR is mostly influenced by the size and cellular activity of organs responsible for energy acquisition and processing of food, such as liver, kidney, heart and the alimentary tract (Wiersma et al. 2012; Swanson et al. 2017), whereas variation in maximal energy expenditure for thermogenesis is primarily influenced by the overall size, size of individual muscle fibres and cellular activity of the pectoralis muscles, which are ultimately responsible for shivering thermogenesis (Jimenez and Williams 2014; Petit and Vézina 2014b). Although, the size and activity of these systems often vary in concert in relation to season (Zheng et al. 2014a), BMR has been documented to respond faster to changes in ambient temperature (Dubois et al. 2016) and show a different temporal adjustment to seasonal changes (Petit et al. 2013) than maximal energy expenditure for thermogenesis. Furthermore, both BMR (Rønning et al. 2015; Nilsson and Nilsson 2016) and maximal energy expenditure for thermogenesis (Petit et al. 2017; Latimer et al. 2018) have been documented to influence winter survival.

Intra-specific variation in BMR depends on both state-dependent and environmental factors. BMR is partly a genetically determined trait that remains individually repeatable (Versteegh et al. 2008; Broggi et al. 2009) with moderate but significant heritability in studies of passerine birds (Rønning et al. 2007; Nilsson et al. 2009; Tieleman et al. 2009; Bushuev et al. 2012; Mathot et al. 2013). However, it is also a labile trait (i.e. phenotypically flexible) that can change reversibly with environmental and ecological circumstances (see McKechnie (2008) and Swanson (2010) for reviews). Previous intra-specific studies have reported BMR plasticity within individuals, resulting in variation among populations in responses to prevailing conditions, including photoperiod (Broggi et al. 2019) and ambient temperature (e.g. Swanson and Olmstead 1999; McKechnie et al. 2007; Petit and Vézina 2014a; Broggi et al. 2019). Ambient temperature also proved to be the main driver explaining inter-specific variation in BMR in rodents (Naya et al. 2013).

After statistically accounting for responses to prevailing environmental conditions during winter, differences in BMR between winters often remain significant (Broggi et al. 2019). This inter-annual variation has been interpreted as originating from stochastic variation (Bouwhuis et al. 2011), although it might also be indicative of cohort effects (Swanson and Olmstead 1999; Broggi et al. 2019). Conditions during early development can have a crucial influence on the adult phenotype (Reid et al. 2003), either influencing the developing offspring directly, or through indirect effects mediated through parental performance, i.e. transgenerational plasticity (Beaman et al. 2016). Thus, shared environmental conditions during development may result in phenotypic uniformity among young in synchronously breeding populations. Such cohort effects have been demonstrated in passerines, for example in the red-billed chough *Pyrrhocorax pyrrhocorax* (Reid et al. 2003), but primarily in three well-studied ungulate populations (Rose et al. 1999; Forchhammer et al. 2001; Douhard et al. 2013), with potential effects on population dynamics (Lindström and Kokko 2002).

The mechanistic causes underlying cohort effects, arising during early developmental stages and manifested as variations in growth rate or survival probability, are largely unexplored. Among the potential traits affected by early-life conditions, telomere shortening has been suggested to be important (Fairlie et al. 2016), although many other morphological and physiological traits may be susceptible to non-reversible developmental plasticity, with long-term fitness consequences (Monaghan 2008). Thus, such traits could pre-determine, for example, the adult metabolic phenotype because of conditions experienced during specific developmental stages (West-Eberhard 2003; McKechnie and Swanson 2010). In avian systems, ambient temperature stands out among environmental drivers, as it can influence the developing offspring directly during the pre- and post-hatching stages, and indirectly as influenced by parental performance during egg laying, incubation and brooding (Careau et al. 2014; Beaman et al. 2016). However, the long-term consequences of such early conditioning, and the precise effect of the environmental/physiological drivers remain poorly understood, particularly in wild animal populations.

We studied the among-year variation in winter BMR of first-year, wild great tits *Parus major*. Cohort effects might be particularly influential in short-lived, small passerines, as these populations are composed by a large proportion of first-year birds. We first calculated annual population means of BMR while controlling for date, the corresponding environmental factors and individual mass. We then used these annual means to test the influence of ambient temperatures during different developmental stages and predicted that the early-life conditions would affect the standardized BMR during their first winter. First, cold ambient temperatures during the egg laying stage may reduce parental investment in eggs (Ojanen 1983). Smaller eggs, or eggs produced by females in poor condition have been found to contain higher levels of androgens (Groothuis et al. 2005), resulting in increased growth rate and begging behaviour (Reed and Clark 2011), with a concomitant increase in nestling metabolism (Bachman and Chappell 1998; Jimenez 2018). Thus, a potential effect on the metabolic phenotype during this stage would be mediated by maternal effects. Second, suboptimal incubation temperatures have been shown to increase BMR of developing nestlings (Nord and Nilsson 2011). In this case, the effect would be directly dependent on the temperature realised by the embryos, which would be modulated by female incubation behaviour. Finally, cold ambient temperatures during the nestling phase may reduce parental investment and induce nestling developmental stress, leading to increased BMR (Criscuolo et al. 2008; Careau et al. 2014). Thus, the effect during this stage would mostly be mediated by parental effects in relation to nestling feeding. If these increases in nestling and juvenile metabolism are irreversible, we predict that cold ambient temperatures during the egg laying, incubation and nestling feeding stages, respectively, will increase BMR during their first winter.

## Materials and methods

### Study area and birds

We measured winter BMR of wild, first-year great tits in Oulu (Finland) (65° N, 25° 30′ E), over a period of 11 winters from 1999 to 2000 until 2005–2006, 2007–2008 and from 2011 to 2012 until 2013–2014 from mid-October to mid-April (median 16 January). The Oulu study area is composed of boreal forests dominated by conifers, Scots pine *Pinus sylvestris* and spruce *Picea abies*, and diverse birch *Betula sp.* species. Winter conditions in the Oulu area are influenced by the high latitude, with minimum temperatures on capture days ranging from -32.0 °C to + 7 °C and day lengths during the peak of mid-winter of 3.5 h.

The great tit is a small-sized passerine (19.2 g ± 1.53) that inhabits temperate forests across Eurasia. In our study area, great tits start egg laying during mid-May, females incubate alone while both parents feed the nestlings. During winter months, great tits invariably move from the breeding territories and join loose flocks near human settlements. The species is actively spreading northwards through Fennoscandia, with the studied population in the Oulu region being close to the northernmost margin of the distribution (Valkama et al. 2011). Boreal conditions are suboptimal for the species, which result in low and temporally fluctuating juvenile survival rates likely as a consequence of harsh winter conditions (Karvonen et al. 2012). Additionally, as many other passerine species at northern latitudes, the Oulu population is time-constrained by the short breeding season, which promotes synchronous breeding (Pakanen et al. 2016).

**Fig. 1:**
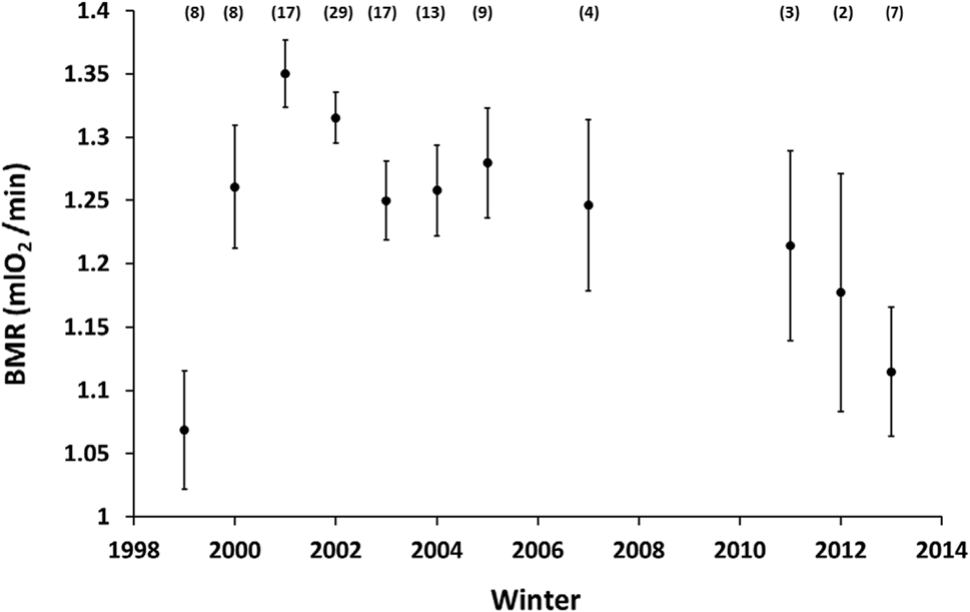
Least square means (± SE) of BMR of first-year great tits during 11 winters when standardized for the factors in Table 2, i.e. date, minimum temperature and body mass. Winter denotes the year when the winter started, thus 1999 = the winter of 1999–2000. Numbers in parentheses denotes sample size

Birds were captured in the vicinity of the University campus soon before dusk at baited funnel traps and weighed (to the nearest 0.1 g). Since few individuals in our winter population are recaptures from the studied breeding population located 10 km to the north, birds were sexed and aged (as first-year or older) by plumage characteristics (Jenni and Winkel 1994), individually marked and brought to the university facilities for BMR measurements (see below). In total, we captured 117 first-year individuals from which we obtained 160 BMR measurements, with up to 5 recaptures of the same individual within a winter. During each winter, we measured between 2 and 29 first-year individuals (Fig. 1). Fifteen first-year great tits were also captured in subsequent winters (12 individuals during 2 and 3 during 3 consecutive winters) making it possible to calculate between-year repeatability. More details on the study area and trapping procedures are provided elsewhere (Broggi et al. 2004).

### Metabolic measurements

BMR is defined as the average minimal oxygen consumption under post-absorptive digestive conditions during the resting phase of the daily cycle of non-growing, non-reproductive endotherms at thermoneutrality (McNab 1997). Thus, BMR was measured as the lowest 5-min running averages of oxygen consumption during the night, in an open-circuit respirometer in a dark climate cabinet, at a constant temperature of 25 °C (mean ± S.D. for a typical night: 24.8 ± 1.65) that is within the species thermoneutral zone (Reinertsen and Haftorn 1986). In short, outdoor dry air was pushed through four independent mass-flow controllers (FMA-A2407, Omega Engineering, Inc. (USA)) at 300 ml/min into plexiglass metabolic chambers (1,6 L) containing an individual bird. Constantly flowing incoming and outcoming air was scrubbed for CO_2_ and H_2_O, with sodalime and silica scrubbers, then conveyed through a multiplexer that directed air from each channel in turns of 12 min to the Oxygen analyzer (S-3A Ametek (USA)). Each cycle lasted an hour and included a fifth channel with reference air to control for the analyzer bias, making it possible to measure a maximum of four birds simultaneously per night. Recorded data were visually inspected and converted to mL O_2_/min following Hill (1972). Most measurement sessions (80%) started between 15:00 and 18:00 (range 15:00–21:00) and BMR estimates (the lowest 5-min running averages of oxygen consumption) were invariably recorded between midnight and 04:00, ensuring post-absorptive state of all individuals (own unpubl. data). After the overnight measurements, birds were released at the point of capture. All procedures were conducted in agreement with the local and national ethical committees.

### Environmental winter and breeding data

In a first set of analyses, we studied the effects of the regional and local winter environment on winter BMR of the first-year birds. Date (October 1st = 1) was included as a linear factor to control for any somatic or physiological effects on BMR that may have been accumulated progressively since the start of the winter (Swanson 2010). Furthermore, we also included day length (time between sun rise and sun set) to represent the time available for feeding and night-time forced fasting (Carey and Dawson 1999; Broggi et al. 2019). We included the North Atlantic Oscillation (NAO) index (obtained from https://www.cpc.ncep.noaa.gov/products/precip/CWlink/pna/nao.shtml) as a measure of large-scale (regional) climatic effects (calculated as the monthly average prior to each individual’s capture date). Positive NAO index is generally associated with milder and moister winter conditions at higher latitudes in Western Europe. Furthermore, to evaluate the effect of local conditions, we incorporated the average minimum ambient temperature (°C) that reflects thermoregulatory costs, and the atmospheric pressure (mBAR) that can be used as a proxy of precipitation (Carey and Dawson 1999). These local conditions were estimated for each capture dates over three different time periods, namely immediate (day of capture), previous short-term (daily average of the week before capture) and previous long-term (daily average of the month before capture) environmental conditions from the date of capture.

In a second set of analyses, we studied the effect of ambient temperature during early development on the metabolic phenotype of great tits. We estimated population average breeding parameters from the neighbouring nest-box study population (10 km north of the winter trapping area) that has been continuously monitored over decades (Karvonen et al. 2012; Pakanen et al. 2016; Vatka et al. 2016). Here, we used annual average date of first egg (77–141 estimates per year), clutch size (75–148 clutches per year) and hatching date (63–118 estimates per year) to define three developmental periods for each year of the study. The periods considered were defined as: Egg laying: laying of the first egg + clutch size; Incubation: from 14 days previous to hatching date until hatching date [from estimates of the incubation length in the same population see Pakanen et al. (2016)]; Nestling provisioning: hatching date + 21 days (based on an average nestling period of 18 days from estimations in the same population (Orell and Ojanen 1983) plus 3 days as fledglings when they potentially are susceptible to environmental conditions. For these time periods, we extracted daily average and minimum temperatures for the year when each measured first-year great tit was born. Atmospheric pressure and ambient temperatures were obtained from the climate station Oulu Linnanmaa situated 4 km south of the trapping area.

### Statistical analyses

All analyses were performed in SAS Enterprise Guide 7.1 (SAS Institute Inc., Cary, NC, USA). To evaluate the effects of the minimum ambient temperature (°C) and atmospheric pressure (mBAR), we first tested which of the three time periods (immediate, previous short-term and previous long-term) that best explained the observed variation. Since the three measures of temperature and atmospheric pressure are interdependent, the effects were included separately together with body mass as a covariate, and their fit was evaluated by Akaike’s information criterion (AIC). Although the AICs were rather similar, the period with the best fit for both the average minimum temperature and for the average atmospheric pressure was the week before BMR measurements (previous short-term) and these two factors were included in the model of within-winter effects on BMR (Table S1).

In a first set of analyses, we evaluated the effect of intrinsic factors of the individuals (sex and body mass) together with prevailing environmental conditions (NAO index, date, day length, winter, minimum temperature, and atmospheric pressure) on winter BMR. We evaluated these factors by using general linear mixed models fitted with maximum-likelihood and selected the best models according to AIC. We included winter (winter 1999–2000 = 1) and sex (0 = male and 1 = female) as categorical predictors and individual identity as a random factor, while body mass, NAO index, date, day length as well as short-term average minimum temperature and atmospheric pressure were considered as continuous predictors. To accommodate different effects of day length in early and late parts of the winter season, we also included the interaction between date and day length. To test the biological significance of the selected factors, we included these factors in a general linear mixed model fitted with the restricted maximum-likelihood method. This model was used in a second set of analyses, to extract yearly standardized means from a model including the selected intrinsic and winter environmental factors to study among-winter variation in BMR. We used the predicted values from this model to explain within-winter sources of variation, thus being the dependent variable in the following analyses. To investigate the potential influence of ambient temperature during early life on the winter metabolic phenotype of first-year great tits, we performed two sets of analyses; one with the average daily temperature during the egg laying, the incubation and the nestling phases, and one where average mean temperature was exchanged for average minimum temperature. Variance inflation factors (VIFs) for the temperature estimates within each analysis were only moderately collinear as VIFs were < 2.5, thus below the level VIF > 3 when collinearity could bias analyses (Zuur et al. 2010). These two models were fitted with the temperature measurements during the three developmental periods and average laying date, to accommodate potential variation due to high-quality females starting to breed early, as covariates and the best model was selected according to AICc. We estimated degrees of freedom by the Satterthwaite method.

We also tested for a potential association between average minimum temperature during spring and the average daily minimum temperatures during January and February the succeeding winter. Repeatabilities were calculated by a one-way ANOVA according to Lessells and Boag (1987). When individuals were measured more than once within a winter, we selected one estimate per year to achieve a time period between measurements as close to 365 days as possible (cf. Broggi et al. 2009; Cortés et al. 2015). All *p* values are two-tailed. All continuous variables and model residuals fulfilled the requirements of normality.

## Results

Average winter BMR was 1.28 ± 0.18 mL O_2_/min. Of the factors that could be predicted to have a direct effect on the winter BMR of first-year great tits (intrinsic, climatic and date factors), 4 were included in all 12 competing models (ΔAIC < 2) (Table 1; for a list of all models see Supplementary Table S2). These factors; body mass, winter, date and minimum ambient temperatures also explained a significant part of the variation in BMR (Table 2). BMR decreased linearly as winter progressed (Fig. S1) and increased with decreasing minimum ambient temperatures (Fig. S2), while it increased with body mass (Table 2). However, even when accounting for these sources of variation, we still found substantial among-winter variation not explained by the other explanatory variables (Table 2; Fig. 1).

**Table 1:** All competing models (ΔAIC < 2) for explaining BMR (ml O_2_/min) in first-year great tits during winter

Model	AIC
Body mass + Min. temp. + Date + Winter + Daylength + Date × Daylength	− 184.7
Body mass + Min. temp. + Date + Winter + Daylength + NAO + Date × Daylength	− 184.5
Body mass + Min. temp. + Date + Winter	− 184.3
Body mass + Min. temp. + Date + Winter + Daylength	− 184.1
Body mass + Min. temp. + Date + Winter + Daylength + Sex + Date × Daylength	− 184.1
Body mass + Min. temp. + Date + Winter + Daylength + NAO + Sex + Date × Daylength	− 183.7
Body mass + Min. temp. + Date + Winter + Daylength + Sex	− 183.1
Body mass + Min. temp. + Date + Winter + Sex	− 183.1
Body mass + Min. temp. + Date + Winter + Daylength + NAO	− 183.0
Body mass + Min. temp. + Date + Winter + NAO	− 183.0
Body mass + Min. temp. + Date + Winter + Atm pressure	− 182.8
Body mass + Min. temp. + Date + Winter + Daylength + Atm pressure + Date × Daylength	− 182.7

**Table 2:** Model with the factors selected by AIC explaining first-year great tit BMR (ml O_2_/min) during winter

Factor	Estimate (± SE)	*F* value	*df*	*p*
Date	− 0.00093 (0.00027)	12.0	1122	0.0007
Min temperature	− 0.00653 (0.00196)	11.1	1145	0.0011
Winter		4.35	10,129	< 0.0001
Body mass	0.04463 (0.00790)	31.9	1122	< 0.0001

Min temperature refers to the average minimum temperature the week before the metabolic rate measurements. Winter refers to the winter of measurement. Date 1 = October 1st. Continuous variables are presented with estimate (± SE) and all variables with *F* value, *df* and *p* value. *N* = 117 individuals

**Fig. 2:**
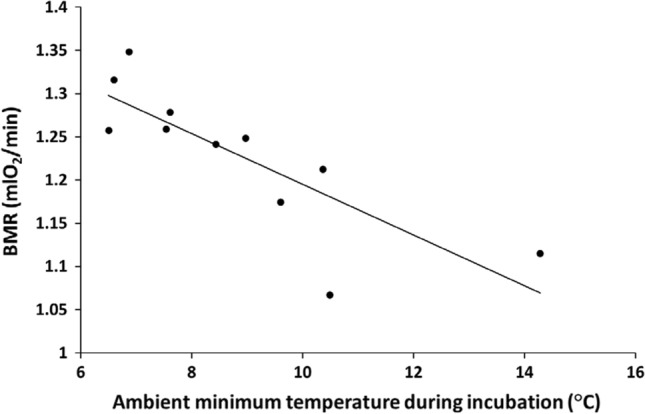
Average winter BMR for first-year great tits, standardized by date, average minimum temperatures the week before capture and body mass at capture, i.e. corrected for the model presented in Table 2. Winter estimates of BMR for first-year birds are presented in relation to minimum temperatures experienced during the incubation period in the previous breeding season (*F*_1,9_ = 17.9, *p* = 0.0022). Equation of the line: BMR = 1.49 – 0.029 × Temp

To account for some of the inter-annual variation in BMR, independently from winter-specific environmental conditions, we explored the effect of conditions experienced during earlier life stages, i.e. during egg laying, incubation and nestling provisioning. We found that only temperatures during the incubation period were included in the best models according to AICc (Table S3). Accordingly, incubation ambient temperature significantly explained the inter-annual variation in population BMR of first-year birds in both sets of temperature models (average temperature: *F*_1,9_ = 11.4, *p* = 0.0081; minimum temperature: *F*_1,9_ = 17.9, *p* = 0.0022). Judging from AICc, the model with minimum temperature performed better than the one with average temperature (*ΔAICc* = 3.0), the two models explaining 67 and 56%, respectively, of the variation in BMR of first-year individuals among winters (Fig. 2). Temperatures during the other two time periods (i.e. egg laying and nestling provisioning periods) had no significant association with winter BMR (Table S2). Furthermore, we found a moderate ability for the average minimum temperature during incubation (but not for the other two periods, *p* > 0.8) to predict the average minimum winter temperature during the following months of January and February at our study site (linear regression: *F*_1,14_ = 6.59, *p* = 0.022, *R*^2^ = 0.32, *slope* =  + 0.89).

Furthermore, since some of the yearly estimates were based on few BMR measurements (Fig. 1), we restricted the analyses of the relation between winter BMR and average ambient temperatures during the different breeding stages, to years in where at least 7 first-year individuals were measured (*N* = 8 winters). As in the analyses of the full data-set, average minimum ambient temperature best explained our variation in winter BMR and this temperature during the incubation phase was the only factor significantly related to BMR of the first-year great tits during winter (*estimate* ± *SE*: − 0.030 ± 0.0084, *F*_1,6_ = 12.7, *p* = 0.012). In addition, BMR was significantly repeatable within individuals between winter seasons (*r* = 0.454, *F*_14,18_ = 2.83, *p* = 0.020).

## Discussion

The winter BMR of first-year great tits was affected by both reversible and non-reversible environmental effects, especially in relation to ambient temperature. Individual winter BMR increased with decreasing minimum temperatures, suggesting a reversible effect of ambient temperature that has also been commonly reported in other studies of tits (Bouwhuis et al. 2011; Petit and Vézina 2014a; Broggi et al. 2019). However, most importantly, when the variation due to winter conditions were accounted for, the minimum ambient temperatures experienced early in life, during the incubation period, explained a significant part of the remaining variation in winter BMR. Low minimum ambient temperatures during incubation resulted in wintering first-year individuals with higher BMR than predicted from e.g. ambient minimum temperatures during the winter (Fig. 2). Furthermore, we found a significant repeatability of BMR between winters (*r* = 0.454) similar to previous between-year repeatabilities calculated on a larger data-set from the same population (*r* = 0.533, Broggi et al. 2009). Thus, first-year birds, incubated during cold conditions, seem to be primed to a high BMR irrespective of encountered winter conditions later in life, as opposed to those incubated during warm springs. This permanent early effect on BMR suggests a form of non-reversible plasticity, due to either a direct effect of ambient temperature on developing embryos, or an indirect effect modulated by maternal incubation behaviour that resulted in long-term effects on the adult metabolic phenotype. We acknowledge that this is a descriptive study and thereby has limited causal predictive power. Experimental incubation of fertilized eggs at different temperatures and monitoring metabolic parameters in subsequent winters would be necessary to provide such a causal link. This was done in blue tits (Nord and Nilsson 2011), although the species has a too low recruitment rate to be able to test the long-term metabolic consequences of different incubation temperatures (Nord and Nilsson 2016).

Incubation entails high levels of heat loss (Mertens 1980) leading to considerable metabolic costs for avian parents in cold environments (Thomson et al. 1998) that can be traded-off against investment in future reproduction (Ardia and Clotfelter 2007). Especially in species with uniparental incubation as in the great tit, egg temperature is necessarily influenced by prevailing ambient temperatures (Ardia and Clotfelter 2007; Ardia et al. 2009). In cold conditions, reduction in egg temperature results in longer incubation periods (Järvinen 1990; Martin et al. 2018), even under increased incubation effort (Nord et al. 2010). Furthermore, suboptimal egg temperatures during incubation are known to impinge on nestling phenotype, with short-term effects on the thermogenic costs of thermoregulation (DuRant et al. 2012) and BMR (Nord and Nilsson 2011; Wada et al. 2015) as well as on survival in captivity (Berntsen and Bech 2016). We found evidence for long-term effects of varying temperatures during development in the egg on adult metabolic phenotype that seem to be non-reversible judging from the significant repeatability between winters. Thus, the thermal environment experienced by the embryos during incubation could promote a transgenerational plastic effect leading to metabolic differences among cohorts.

Conditions experienced during early development, either during the nestling phase (Verhulst et al. 2006; Criscuolo et al. 2008) or during the embryonic stage (Ben-Ezra et al. 2017; this study), may pre-determine the adult metabolic phenotype. There is an unfortunate paucity of studies on potential mechanisms for such a priming of the metabolic phenotype (Nord and Giroud 2020). One candidate mechanism might be temperature-induced epigenetic adaptations, e.g. varying levels of DNA methylation as found in zebra finches exposed to different ambient temperatures during early life (Sheldon et al. 2020). A potential specific mechanism might be epigenetic effects during the ontogeny of the hypothalamus-pituitary-thyroid (HPT) axis (Tzschenke 2008; Loyau et al. 2014; Ruuskanen et al. 2021). The target for epigenetic alterations may be the production of thyroid hormones (especially triiodothyronine, T_3_), demonstrated to increase in cold environments (Zheng et al. 2014b). Increased levels of T_3_ is known to increase thermogenesis and to simultaneously increase BMR (Vézina et al. 2009; Zheng et al. 2014b; Ruuskanen et al. 2021). This mechanism is based on alterations during ontogeny, i.e. during the incubation stage. The fact that we did not find any effects of ambient temperature during the egg laying and nestling stages on adult BMR is compatible with this mechanism. Previous demonstrations of environmental effects originating during the egg laying and nestling stages are based on low resource levels either in the egg or during nestling feeding. The variation in ambient spring temperatures in our study area might not have been sufficient to result in large enough variation in resource levels to affect the metabolic phenotype.

These mechanisms might help the embryo to acclimatize to suboptimal immediate thermal conditions without any positive or even detrimental consequences later in life. However, it could also be an example of adaptive transgenerational plasticity that could improve the success of the individual in the environment it will likely encounter in future life (Monaghan 2008; Ruuskanen et al. 2021). In line with this, Muscovy ducklings *Cairina moschata* originating from eggs incubated at a low temperature preferred lower ambient temperatures after hatching than normally incubated ducklings (Tzschentke 2008). If the temperature perceived by the embryo during incubation is indicative of the general climate during the winter, as found in this study, it could be adaptive to adjust the metabolic phenotype to these predicted conditions. We would predict this effect to be strongest among short-lived, resident species as those are the ones with the largest chance to experience correlated ambient temperatures between the incubation phase and their winter environment. Decreasing ambient temperatures may select for a higher thermogenic capacity with a concomitant increase in BMR (Swanson and Olmstead 1999). This would be beneficial in cold environments, as reflected in typical increases of BMR with latitudinal gradients (Broggi et al. 2019), with the approaching winter season (Swanson 2010), with decreasing ambient temperatures (this study) and a differential survival advantage of high-BMR blue tits in cold environments (Nilsson and Nilsson 2016). Thus, some of the inter-population variation in metabolic rates due to latitude could be set by adaptive developmental plasticity triggered during the incubation phase.

In years when winter ambient temperatures are colder or warmer than expected based on incubation temperatures, we predict a reduced recruitment rate due to a mismatch between ambient conditions and the metabolic phenotype that increases mortality during the winter. Environmental stochasticity impacting on recruitment rate has been suggested as a potential explanation for inter-specific variation in population variability (Sæther et al. 2016). A mismatch in temperatures between the incubation stage and the subsequent winter may constitute such an environmental stochasticity. One of the predictions concerning future climate change is that the unpredictability will increase (IPCC 2012) which potentially will increase the risk of a mismatch between temperatures during spring and winter, resulting in increased variation in population size.

The metabolic phenotype is flexible and can be adjusted to prevailing conditions allowing organisms to buffer changes. However, part of this flexibility seems also to be tuned by the thermal environment experienced during early life. The extent to which temperature conditions change congruently over different individual life-history episodes will determine the adaptive value of this mechanism.

## Supplementary Information

Below is the link to the electronic supplementary material.

Supplementary file1 (DOCX 111 KB)

## Data Availability

Dataset analysed in this study will be available from the Digital CSIC Repository http://dx.doi.org/10.20350/digitalCSIC/12612 upon reasonable request.
