# Supplementary material for: Prehatching temperatures drive inter-annual cohort differences in great tit metabolism
Source: Oecologia. 2022 Feb 17;198(3):619–27. doi: 10.1007/s00442-022-05126-7 (PMC8956552; doi:10.1007/s00442-022-05126-7)

Supplementary information for the article entitled “PREHATCHING TEMPERATURES DRIVE INTERANNUAL COHORT DIFFERENCES IN GREAT TIT METABOLISM” by Juli Broggi^1,2,3^, Esa Hohtola^4^, Kari Koivula^4^, Seppo Rytkönen^4^ and Jan-Åke Nilsson^1^

1 Department of Biology, Section of Evolutionary Ecology, University of Lund, S-223 62 Lund, Sweden

2 Estación Biológica de Doñana (CSIC), Av. Américo Vespucio 26, 41092 Sevilla, Spain.

3 Departamento de Ecología Evolutiva, Museo Nacional de Ciencias Naturales - CSIC, C/José Gutiérrez Abascal 2, Madrid. 28006, Spain

4 Ecology and Genetics Research Unit, University of Oulu, P.O. Box 3000, FIN-90014 Oulu, Finland

Author for correspondace: Juli Broggi Phone: +34629331951; e-mail: julibroggi@gmail.com

Table S1. Two separate models including average minimum temperature and average atmospheric pressure, respectively, during three time periods (the day of capture, the average of the week before capture and the average of the month before capture) explaining BMR (ml O_2_/min) of first-year great tits during winter

| **Model** | **AIC** |
| --- | --- |
|  |  |
| **Average minimum temperature** |  |
|  |  |
| Body mass + Day of capture | -146.9 |
| Body mass + Week before capture | -150.1 |
| Body mass + Month before capture | -148.6 |
|  |  |
| **Average atmospheric pressure** |  |
|  |  |
| Body mass + Day of capture | -147.2 |
| Body mass + Week before capture | -148.5 |
| Body mass + Month before capture | -146.6 |
|  |  |

Table S2. All competing models for explaining BMR (ml O_2_/min) of first-year great tits during winter ordered according to decreasing AIC. The top models (ΔAIC < 2) in bold.

| **Model** | **AIC** |
| --- | --- |
|  |  |
| **Body mass + Min. temp. + Date + Winter + Daylength + Date × Daylength** | **-184.7** |
| **Body mass + Min. temp. + Date + Winter + Daylength + NAO + Date × Daylength** | **-184.5** |
| **Body mass + Min. temp. + Date + Winter** | **-184.3** |
| **Body mass + Min. temp. + Date + Winter + Daylength** | **-184.1** |
| **Body mass + Min. temp. + Date + Winter + Daylength + Sex + Date × Daylength** | **-184.1** |
| **Body mass + Min. temp. + Date + Winter + Daylength + NAO + Sex + Date × Daylength** | **-183.7** |
| **Body mass + Min. temp. + Date + Winter + Daylength + Sex** | **-183.1** |
| **Body mass + Min. temp. + Date + Winter + Sex** | **-183.1** |
| **Body mass + Min. temp. + Date + Winter + Daylength + NAO** | **-183.0** |
| **Body mass + Min. temp. + Date + Winter + NAO** | **-183.0** |
| **Body mass + Min. temp. + Date + Winter + Atm pressure** | **-182.8** |
| **Body mass + Min. temp. + Date + Winter + Daylength + Atm pressure + Date × Daylength** | **-182.7** |
| Body mass + Min. temp. + Date + Winter + Daylength + NAO + Atm pressure + Date × Daylength | -182.5 |
| Body mass + Min. temp. + Date + Winter + Daylength + Atm pressure | -182.5 |
| Body mass + Winter + Daylength + NAO | -182.3 |
| Body mass + Min. temp. + Date + Winter + Daylength + Atm pressure + Sex + Date × Daylength | -182.1 |
| Body mass + Min. temp. + Winter + Daylength | -182.1 |
| Body mass + Min. temp. + Date + Winter + Daylength + NAO + Sex | -181.9 |
| Body mass + Min. temp. + Date + Winter + Daylength + NAO + Atm pressure + Sex + Date × Daylength | -181.7 |
| Body mass + Winter + Daylength + Sex | -181.7 |
| Body mass + Min. temp. + Date + Winter + Daylength + Atm pressure + Sex | -181.6 |
| Body mass + Min. temp. + Date + Winter + NAO + Sex | -181.6 |
| Body mass + Min. temp. + Date + Winter + Atm pressure + Sex | -181.6 |
| Body mass + Min. temp. + Winter + Daylength + NAO | -181.6 |
| Body mass + Winter + Daylength + NAO + Sex | -181.6 |
| Body mass + Min. temp. + Date + Winter + Daylength + NAO + Atm pressure | -181.5 |
| Body mass + Min. temp. + Date + Winter + NAO + Atm pressure | -181.5 |
| Body mass + Winter + Daylength | -181.5 |
| Body mass + Min. temp. + Winter + Daylength + Sex | -181.2 |
| Body mass + Date + Winter + Daylength + NAO + Sex + Date × Daylength | -181.0 |
| Body mass + Date + Winter + Daylength + NAO + Date × Daylength | -181.0 |
| Body mass + Date + Winter + Daylength + NAO | -180.8 |
| Body mass + Min. temp. + Winter + Daylength + Atm pressure | -180.6 |
| Body mass + Winter + Daylength + NAO + Atm pressure | -180.6 |
| Body mass + Min. temp. + Date + Winter + Daylength + NAO + Atm pressure + Sex | -180.5 |
| Body mass + Min. temp. + Date + Winter + Daylength + NAO + Sex | -180.5 |
| Body mass + Date + Winter + Daylength + Sex | -180.4 |
| Body mass + Date + Winter + Daylength | -180.4 |
| Body mass + Date + Winter + Daylength + NAO + Sex | -180.3 |
| Body mass + Min. temp. + Winter + Daylength + NAO + Atm pressure | -180.2 |
| Body mass + Winter + Daylength + NAO + Atm pressure + Sex | -180.1 |
| Body mass + Winter + Daylength + Atm pressure | -180.1 |
| Body mass + Winter + Daylength + Atm pressure + Sex | -180.0 |
| Body mass + Date + Winter + Daylength + Sex + Date × Daylength | -179.9 |
| Body mass + Min. temp. + Winter + Daylength + Atm pressure + Sex | -179.8 |
| Body mass + Date + Winter + Daylength + Date × Daylength | -179.4 |
| Body mass + Date + Winter + Daylength + NAO + Atm pressure + Sex + Date × Daylength | -179.0 |
| Body mass + Date + Winter + Daylength + NAO + Atm pressure + Date × Daylength | -179.0 |
| Body mass + Date + Winter + Daylength + NAO + Atm pressure | -179.0 |
| Body mass + Date + Winter + Daylength + NAO + Atm pressure + Sex | -178.7 |
| Body mass + Date + Winter + Daylength + Atm pressure + Sex | -178.6 |
| Body mass + Min. temp. + Date + Winter + NAO + Atm pressure + Sex + Date × Daylength | -178.5 |
| Body mass + Date + Winter + Daylength + Atm pressure | -178.5 |
| Body mass + Min. temp. + Winter + Daylength + NAO + Atm pressure + Sex + Date × Daylength | -178.3 |
| Body mass + Date + Winter + Daylength + Atm pressure + Sex + Date × Daylength | -177.9 |
| Body mass + Date + Winter + Daylength + Atm pressure + Date × Daylength | -177.4 |
| Body mass + Date + Winter +NAO | -175.1 |
| Body mass + Date + Winter | -174.6 |
| Body mass + Date + Winter + NAO + Sex | -174.6 |
| Body mass + Date + Winter + Sex | -174.6 |
| Body mass + Date + Winter | -174.6 |
| Body mass + Min. temp. + Winter | -174.5 |
| Body mass + Min. temp. + Winter + NAO | -173.8 |
| Body mass + Date + Winter + NAO + Atm pressure | -173.2 |
| Body mass + Min. temp. + Winter + Sex | -173.1 |
| Body mass + Min. temp. + Winter + Atm pressure | -173.0 |
| Body mass + Date + Winter + NAO + Atm pressure + Sex | -172.8 |
| Body mass + Winter + NAO | -172.7 |
| Body mass + Date + Winter + Atm pressure + Sex | -172.6 |
| Body mass + Date + Winter + Atm pressure | -172.6 |
| Body mass + Min. temp. + Winter + NAO + Atm pressure | -172.4 |
| Body mass + Winter | -172.3 |
| Body mass + Min. temp. + Winter + NAO + Sex | -172.2 |
| Body mass + Min. temp. + Winter + Atm pressure + Sex | -171.6 |
| Body mass + Winter + NAO + Sex | -171.6 |
| Body mass + Winter + Sex | -171.6 |
| Body mass + Winter + NAO + Atm pressure | -171.0 |
| Body mass + Min. temp. + Winter + NAO + Atm pressure + Sex | -170.9 |
| Body mass + Winter + Atm pressure | -170.4 |
| Body mass + Winter + NAO + Atm pressure + Sex | -169.9 |
| Body mass + Winter + Atm pressure + Sex | -169.8 |
| Body mass + Min. temp. + Date + Daylength + Sex + Date × Daylength | -166.0 |
| Body mass + Min. temp. + Daylength + Sex | -165.8 |
| Body mass + Min. temp. + Daylength + Atm pressure + Sex | -165.7 |
| Body mass + Min. temp. + Date + Daylength + Sex | -165.6 |
| Body mass + Min. temp. + Date + Daylength + Atm pressure + Sex | -164.8 |
| Body mass + Min. temp. + Date + Daylength | -164.6 |
| Body mass + Min. temp. + Daylength + Atm pressure | -164.5 |
| Body mass + Min. temp. + Date + Daylength + Date × Daylength | -164.4 |
| Body mass + Min. temp. + Daylength | -164.4 |
| Body mass + Min. temp. + Date + Daylength + Atm pressure + Sex + Date × Daylength | -164.3 |
| Body mass + Min. temp. + Date + Daylength + NAO + Sex + Date × Daylength | -164.1 |
| Body mass + Min. temp. + Daylength + NAO + Atm pressure + Sex | -164.0 |
| Body mass + Min. temp. + Date + Daylength + NAO + Sex | -163.9 |
| Body mass + Min. temp. + Daylength + NAO + Sex | -163.9 |
| Body mass + Min. temp. + Date + Daylength + Atm pressure | -163.7 |
| Body mass + Min. temp. + Date + Sex | -163.5 |
| Body mass + Min. temp. + Date + Daylength + NAO + Atm pressure + Sex | -163.3 |
| Body mass + Min. temp. + Date + Daylength + NAO | -163.1 |
| Body mass + Daylength + Sex | -162.9 |
| Body mass + Min. temp. + Daylength + NAO + Atm pressure | -162.8 |
| Body mass + Min. temp. + Daylength + NAO | -162.8 |
| Body mass + Min. temp. + Date + Daylength + NAO + Date × Daylength | -162.7 |
| Body mass + Min. temp. + Date + Daylength + Atm pressure + Date × Daylength | -162.7 |
| Body mass + Min. temp. + Date + Atm pressure + Sex | -162.7 |
| Body mass + Min. temp. + Date | -162.6 |
| Body mass + Min. temp. + Date + Daylength + NAO + Atm pressure + Sex + Date × Daylength | -162.5 |
| Body mass + Min. temp. + Date + Daylength + NAO + Atm pressure | -162.3 |
| Body mass + Daylength + Atm pressure + Sex | -162.2 |
| Body mass + Min. temp. + Date + NAO + Sex | -161.9 |
| Body mass + Min. temp. + Date + Atm pressure | -161.7 |
| Body mass + Date + Daylength + Sex | -161.6 |
| Body mass + Min. temp. + Date + NAO + Atm pressure + Sex | -161.2 |
| Body mass + Date + Daylength + Atm pressure + Sex | -161.2 |
| Body mass + Min. temp. + Date + NAO | -161.2 |
| Body mass + Min. temp. + Date + Daylength + NAO + Atm pressure + Date × Daylength | -161.1 |
| Body mass + Date + Daylength + Sex + Date × Daylength | -161.1 |
| Body mass + Daylength + NAO + Sex | -160.9 |
| Body mass + Daylength | -160.9 |
| Body mass + Daylength + NAO + Atm pressure + Sex | -160.7 |
| Body mass + Min. temp. + Date + NAO + Atm pressure | -160.4 |
| Body mass + Daylength + Atm pressure | -160.0 |
| Body mass + Date + Daylength + Atm pressure + Sex + Date × Daylength | -159.8 |
| Body mass + Date + Daylength + NAO + Sex | -159.6 |
| Body mass + Date + Daylength | -159.3 |
| Body mass + Date + Daylength + NAO + Atm pressure + Sex | -159.2 |
| Body mass + Date + Daylength + NAO + Sex + Date × Daylength | -159.1 |
| Body mass + Date + Daylength + Atm pressure | -159.1 |
| Body mass + Daylength + NAO | -158.9 |
| Body mass + Daylength + NAO + Atm pressure | -158.5 |
| Body mass + Date + Daylength + Date × Daylength | -158.2 |
| Body mass + Date + Daylength + NAO + Atm pressure + Sex + Date × Daylength | -157.8 |
| Body mass + Date + Daylength + NAO | -157.7 |
| Body mass + Date + Daylength + Atm pressure + Date × Daylength | -157.4 |
| Body mass + Date + Daylength + NAO + Atm pressure | -157.1 |
| Body mass + Date + Daylength + NAO + Date × Daylength | -156.7 |
| Body mass + Min. temp. + Atm pressure + Sex | -155.8 |
| Body mass + Date + Daylength + NAO + Atm pressure + Date × Daylength | -155.4 |
| Min. temp. + Date + Winter + Daylength + NAO + Date × Daylength | -155.1 |
| Min. temp. + Date + Winter + Daylength | -155.0 |
| Min. temp. + Date + Winter | -154.9 |
| Min. temp. + Date + Winter + Daylength + Date × Daylength | -154.7 |
| Min. temp. + Date + Winter + Daylength + Date × Daylength | -154.7 |
| Min. temp. + Date + Winter + Daylength + NAO | -154.4 |
| Min. temp. + Date + Winter + Sex | -154.4 |
| Body mass + Min. temp. + Atm pressure | -154.3 |
| Min. temp. + Date + Winter + Daylength + NAO + Sex + Date × Daylength | -154.2 |
| Min. temp. + Date + Winter + Daylength + Sex | -154.2 |
| Min. temp. + Date + Winter + NAO | -154.0 |
| Body mass + Min. temp. + Sex | -153.9 |
| Body mass + Min. temp. + NAO + Atm pressure + Sex | -153.8 |
| Min. temp. + Date + Winter + Daylength + NAO + Sex | -153.7 |
| Min. temp. + Date + Winter + Daylength + Sex + Date × Daylength | -153.7 |
| Min. temp. + Date + Winter + NAO + Sex | -153.7 |
| Date + Winter + Daylength + NAO | -153.4 |
| Min. temp. + Date + Winter + Daylength + Atm pressure | -153.2 |
| Min. temp. + Date + Winter + Daylength + NAO + Atm pressure + Date × Daylength | -153.1 |
| Date + Winter + Daylength + NAO + Date × Daylength | -153.1 |
| Min. temp. + Date + Winter + Atm pressure | -153.1 |
| Body mass + Atm pressure + Sex | -152.8 |
| Min. temp. + Date + Winter + Daylength + NAO + Atm pressure | -152.7 |
| Min. temp. + Date + Winter + Daylength + Atm pressure + Date × Daylength | -152.7 |
| Min. temp. + Date + Winter + Atm pressure + Sex | -152.6 |
| Body mass + Date + Sex | -152.5 |
| Body mass + Min. temp. | -152.5 |
| Winter + Daylength + NAO | -152.4 |
| Min. temp. + Date + Winter + Daylength + Atm pressure + Sex | -152.3 |
| Min. temp. + Date + Winter + NAO + Atm pressure | -152.3 |
| Min. temp. + Date + Winter + Daylength + NAO + Atm pressure + Sex + Date × Daylength | -152.2 |
| Body mass + Date + Atm pressure + Sex | -152.2 |
| Date + Winter + Daylength + NAO + Sex | -152.1 |
| Min. temp. + Date + Winter + Daylength + NAO + Atm pressure + Sex | -152.0 |
| Body mass + Min. temp. + NAO + Sex | -152.0 |
| Date + Winter + Daylength | -152.0 |
| Body mass + Sex | -152.0 |
| Min. temp. + Date + Winter + NAO + Atm pressure + Sex | -151.9 |
| Min. temp. + Date + Winter + Daylength + Atm pressure + Sex + Date × Daylength | -151.7 |
| Winter + Daylength + NAO + Sex | -151.7 |
| Date + Winter + Daylength + NAO + Sex + Date × Daylength | -151.6 |
| Body mass + Min. temp. + NAO + Atm pressure | -151.6 |
| Date + Winter + Daylength + NAO + Atm pressure | -151.5 |
| Winter + Daylength | -151.4 |
| Date + Winter + Daylength + NAO + Atm pressure + Date × Daylength | -151.1 |
| Body mass + NAO + Atm pressure + Sex | -151.0 |
| Date + Winter + Daylength + Sex | -150.9 |
| Winter + Daylength + NAO + Atm pressure | -150.7 |
| Body mass + Date + NAO + Sex | -150.7 |
| Date + Winter + Daylength + Date × Daylength | -150.7 |
| Min. temp. + Winter + Daylength + NAO | -150.5 |
| Date + Winter + Daylength + Atm pressure | -150.5 |
| Body mass + Min. temp. + NAO | -150.5 |
| Body mass + Atm pressure | -150.5 |
| Winter + Daylength + Sex | -150.4 |
| Body mass + Date + NAO + Atm pressure + Sex | -150.3 |
| Date + Winter + Daylength + NAO + Atm pressure + Sex | -150.2 |
| Body mass + Date | -150.1 |
| Min. temp. + Winter + Daylength + NAO + Sex | -150.0 |
| Winter + Daylength + NAO + Atm pressure + Sex | -149.9 |
| Min. temp. + Winter + Daylength | -149.9 |
| Body mass | -149.8 |
| Body mass + Date + Atm pressure | -149.7 |
| Date + Winter + Daylength + NAO + Atm pressure + Sex + Date × Daylength | -149.6 |
| Winter + Daylength + Atm pressure | -149.6 |
| Min. temp. + Winter + Daylength + Sex | -149.3 |
| Date + Winter + Daylength + Atm pressure + Date × Daylength | -149.0 |
| Date + Winter + Daylength + Atm pressure + Sex | -148.9 |
| Min. temp. + Winter + Daylength + NAO + Atm pressure | -148.8 |
| Winter + Daylength + Atm pressure + Sex | -148.5 |
| Min. temp. + Winter + Daylength + NAO + Atm pressure + Sex | -148.4 |
| Min. temp. + Winter + Daylength + Atm pressure | -148.2 |
| Body mass + Date + NAO | -148.2 |
| Body mass + NAO | -148.0 |
| Date + Winter + NAO | -147.8 |
| Body mass + Date + NAO + Atm pressure | -147.7 |
| Min. temp. + Winter + Daylength + Atm pressure + Sex | -147.6 |
| Date + Winter + Daylength + Atm pressure + Sex + Date × Daylength | -147.4 |
| Date + Winter | -146.8 |
| Date + Winter + NAO + Sex | -146.6 |
| Date + Winter + NAO + Atm pressure | -145.8 |
| Date + Winter + Sex | -145.3 |
| Date + Winter + Atm pressure | -144.8 |
| Date + Winter + NAO + Atm pressure + Sex | -144.6 |
| Date + Winter + Atm pressure + Sex | -143.3 |
| Min. temp. + Winter + NAO + Sex | -138.8 |
| Winter + NAO | -138.8 |
| Min. temp. + Winter + Sex | -138.4 |
| Winter | -137.9 |
| Min. temp. + Winter + NAO | -137.7 |
| Winter + Sex | -137.7 |
| Min. temp. + Winter | -137.6 |
| Winter + NAO + Atm pressure + Sex | -137.2 |
| Min. temp. + Winter + NAO + Atm pressure + Sex | -137.1 |
| Min. temp. + Winter + Atm pressure + Sex | -136.6 |
| Min. temp. + Winter + NAO + Atm pressure | -136.0 |
| Winter + Atm pressure | -135.9 |
| Min. temp. + Winter + Atm pressure | -135.8 |
| Winter + Atm pressure + Sex | -135.7 |
| Min. temp. + Daylength | -117.8 |
| Min. temp. + Date + Daylength | -117.6 |
| Daylength | -117.6 |
| Min. temp. + Date + Daylength + NAO | -116.3 |
| Min. temp. + Daylength + NAO | -116.0 |
| Min. temp. + Daylength + Atm pressure | -116.0 |
| Min. temp. + Date + Daylength + Atm pressure | -115.9 |
| Min. temp. + Date + Daylength + Sex | -115.9 |
| Min. temp. + Daylength + Sex | -115.9 |
| Min. temp. + Date + Daylength + Date × Daylength | -115.8 |
| Daylength + Atm pressure | -115.7 |
| Date + Daylength | -115.6 |
| Daylength + NAO | -115.6 |
| Daylength + Sex | -115.6 |
| Min. temp. + Date + Daylength + NAO + Sex | -114.4 |
| Min. temp. + Date + Daylength + NAO + Date × Daylength | -114.4 |
| Min. temp. + Date + Daylength + NAO + Atm pressure | -114.3 |
| Min. temp. + Daylength + NAO + Atm pressure | -114.3 |
| Min. temp. + Date + Daylength + Sex + Date × Daylength | -114.1 |
| Min. temp. + Daylength + NAO + Sex | -114.1 |
| Min. temp. + Daylength + Atm pressure + Sex | -114.1 |
| Min. temp. + Date + Daylength + Atm pressure + Sex | -114.0 |
| Min. temp. + Date + Daylength + Atm pressure + Date × Daylength | -114.0 |
| Daylength + Atm pressure + Sex | -113.7 |
| Date + Daylength + Date × Daylength | -113.7 |
| Min. temp. + Date | -112.9 |
| Min. temp. + Date + Daylength + NAO + Atm pressure + Sex | -112.5 |
| Min. temp. + Date + Daylength + NAO + Sex + Date × Daylength | -112.5 |
| Min. temp. + Date + Daylength + NAO + Atm pressure + Date × Daylength | -112.4 |
| Min. temp. + Daylength + NAO + Atm pressure + Sex | -112.4 |
| Min. temp. + Date + Daylength + Atm pressure + Sex + Date × Daylength | -112.1 |
| Date + Daylength + NAO + Sex | -111.7 |
| Date + Daylength + Atm pressure + Sex | -111.7 |
| Daylength + NAO + Atm pressure + Sex | -111.7 |
| Date + Daylength + NAO + Date × Daylength | -111.7 |
| Date + Daylength + Atm pressure + Date × Daylength | -111.7 |
| Date + Daylength + Sex + Date × Daylength | -111.7 |
| Min. temp. + Date + NAO | -111.4 |
| Date + Daylength + NAO + Atm pressure | -111.2 |
| Min. temp. + Date + Sex | -111.0 |
| Min. temp. + Date + Atm pressure | -110.9 |
| Min. temp. + Date + Daylength + NAO + Atm pressure + Sex + Date × Daylength | -110.5 |
| Date + Daylength + NAO + Atm pressure + Date × Daylength | -110.0 |
| Date + Daylength + NAO + Sex + Date × Daylength | -109.9 |
| Min. temp. + Date + NAO + Sex | -109.6 |
| Min. temp. + Date + NAO + Atm pressure | -109.5 |
| Min. temp. + Date + Atm pressure + Sex | -109.1 |
| Date + Daylength + NAO + Atm pressure + Sex + Date × Daylength | -108.0 |
| Min. temp. + Date + NAO + Atm pressure + Sex | -107.7 |
| Date | -103.6 |
| Date + NAO | -101.6 |
| Date + Atm pressure | -101.6 |
| Date + Sex | -101.6 |
| Date + Atm pressure + Sex | -99.6 |
| Min. temp | -98.5 |
| Date + NAO + Atm pressure + Sex | -97.7 |
| Min. temp. + Atm pressure | -97.6 |
| Atm pressure | -97.6 |
| NAO | -96.9 |
| Sex | -96.9 |
| Min. temp. + Sex | -96.7 |
| Min. temp. + NAO | -96.6 |
| Min. temp. + Atm pressure + Sex | -95.8 |
| NAO + Atm pressure | -95.6 |
| Atm pressure + Sex | -95.6 |
| NAO + Sex | -95.0 |
| Min. temp. + NAO + Atm pressure + Sex | -93.8 |
| NAO + Atm pressure + Sex | -93.7 |

Table S3. All competing models for explaining average winter BMR (ml O_2_/min) of first-year great tits ordered according to decreasing AICc. Explanatory variables are average ambient temperature (first model) or average minimum ambient temperature (second model) during the periods egg laying, incubation and nestling. The top model in both sets of analyses have a ΔAICc larger than 2 compared with the next best model. N = 11 winters.

| **Model** | **AICc** |
| --- | --- |
|  |  |
| **Average daily temperature** |  |
|  |  |
| Incubation temp | -24.2 |
| Laying temp + Incubation temp | -19.9 |
| Lay date + Incubation temp | -19.3 |
| Incubation temp + Nestling temp | -19.1 |
| Lay date | -18.0 |
| Laying temp | -17.8 |
| Nestling temp | -17.0 |
| Lay date + Nestling temp | -14.3 |
| Lay date + Laying temp + Incubation temp | -14.2 |
| Laying temp + Nestling temp | -13.9 |
| Lay date + Laying temp | -13.4 |
| Laying temp + Incubation temp + Nestling temp | -12.6 |
| Lay date + Incubation temp + Nestling temp | -12.1 |
| Lay date + Laying temp + Nestling temp | -7.4 |
| Lay date + Laying temp + Incubation temp + Nestling temp | -3.2 |
|  |  |
| **Average minimum temperature** |  |
|  |  |
| Incubation temp | -27.2 |
| Laying temp + Incubation temp | -24.7 |
| Incubation temp + Nestling temp | -22.0 |
| Lay date + Incubation temp | -22.0 |
| Lay date + Laying temp + Incubation temp | -18.1 |
| Lay date | -18.0 |
| Nestling temp | -17.7 |
| Laying temp + Incubation temp + Nestling temp | -17.4 |
| Laying temp | -17.0 |
| Lay date + Nestling temp | -14.9 |
| Lay date + Incubation temp + Nestling temp | -14.7 |
| Laying temp + Nestling temp | -13.8 |
| Lay date + Laying temp | -12.9 |
| Lay date + Laying temp + Nestling temp | -7.5 |
| Lay date + Laying temp + Incubation temp + Nestling temp | -7.1 |
|  |  |

Figure S1. The relation between BMR (mlO_2_/min) of wintering first-year great tits and number of days since 1^st^ of October.


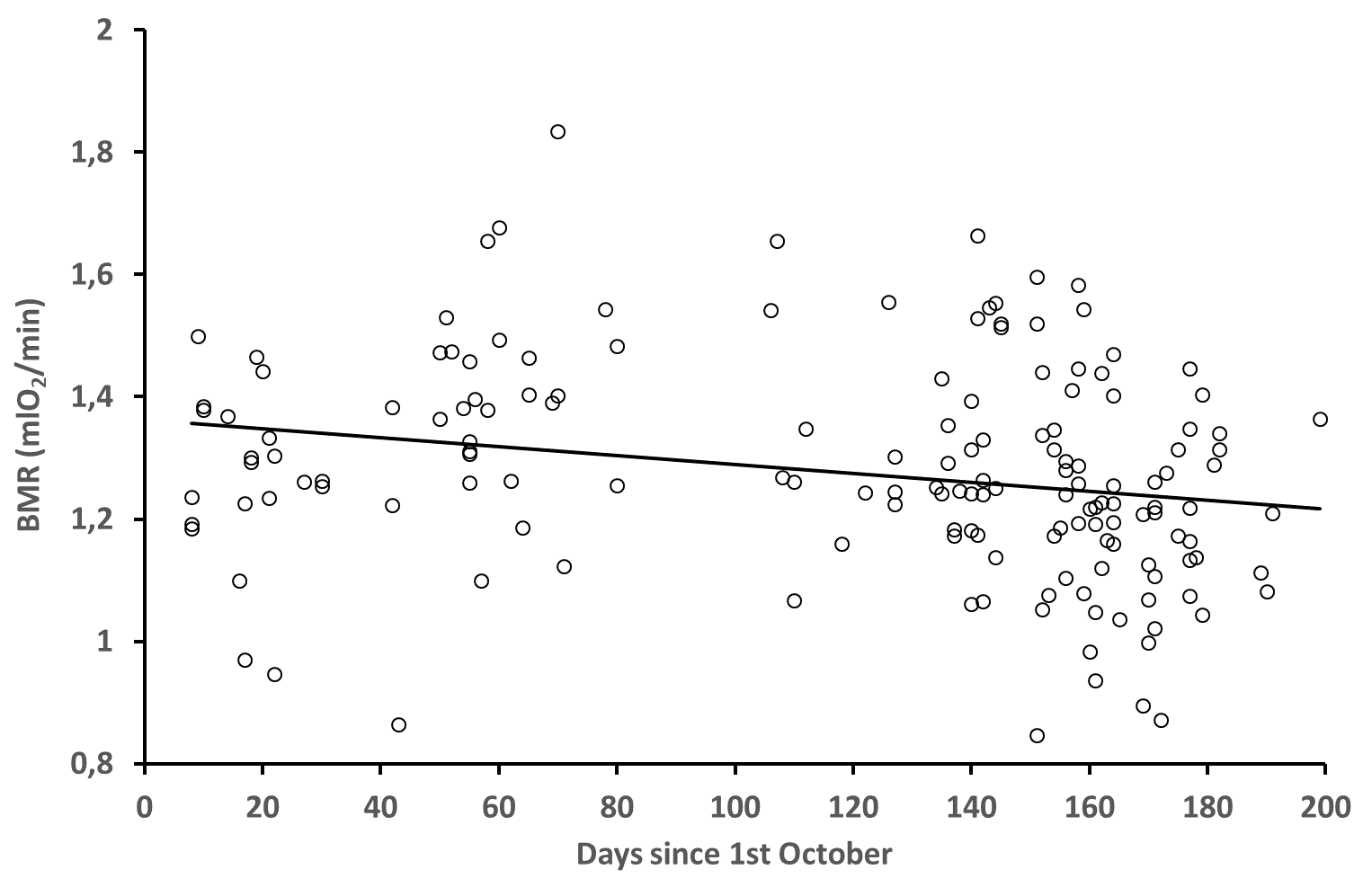


Figure S2. The relation between BMR (mlO_2_/min) of wintering first-year great tits and average minimum temperature (°C) during the week before BMR measurements.


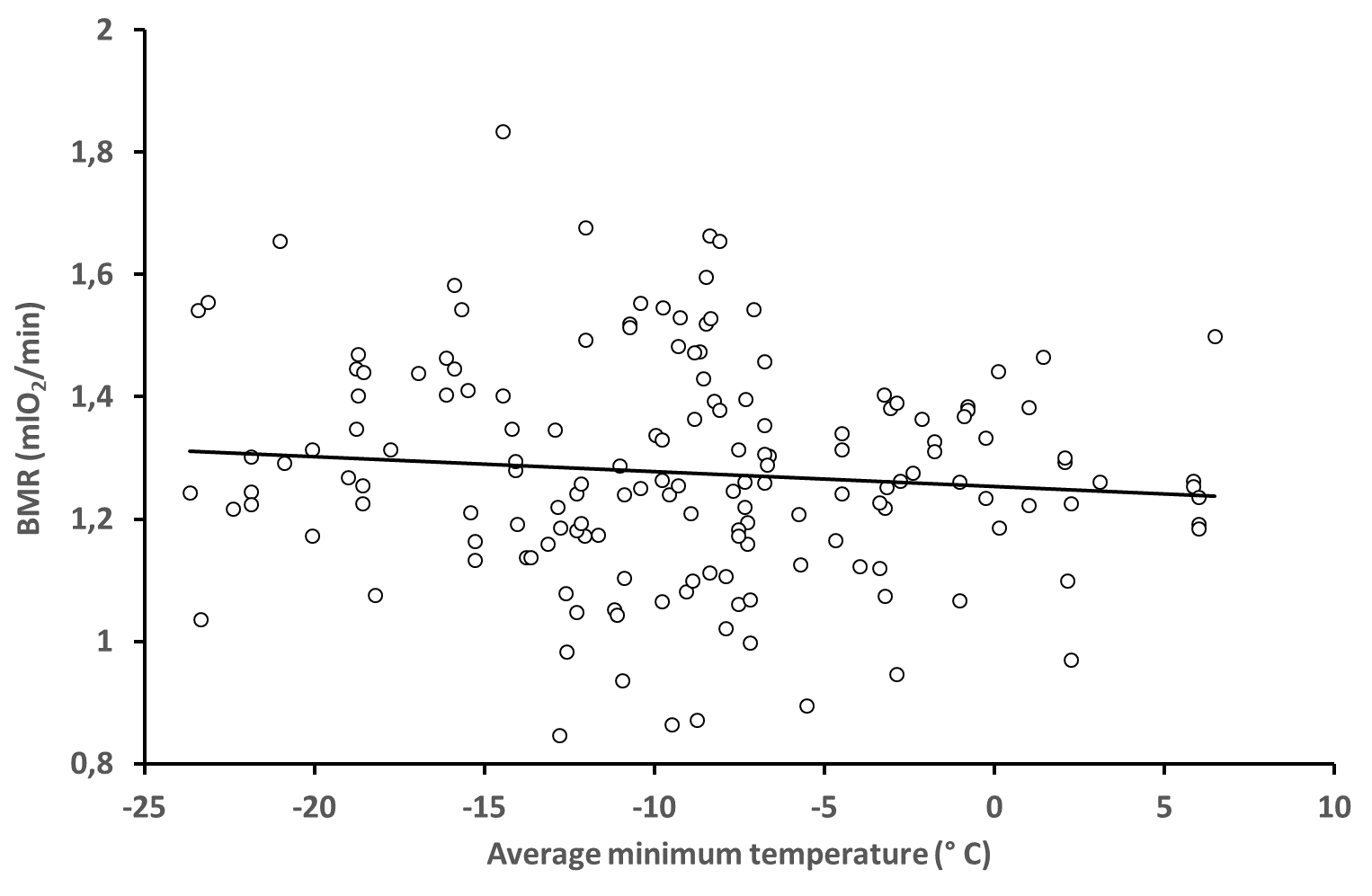

Supplement: Supplementary file 1 — Supplementary file1 (DOCX 111 KB) [file 442_2022_5126_MOESM1_ESM.docx]
